# Dynamic Amyloid and Metabolic Signatures of Delayed Recall Performance within the Clinical Spectrum of Alzheimer’s Disease

**DOI:** 10.3390/brainsci13020232

**Published:** 2023-01-30

**Authors:** Marina Tedeschi Dauar, Tharick Ali Pascoal, Joseph Therriault, Jared Rowley, Sara Mohaddes, Monica Shin, Eduardo R. Zimmer, Simon Fristed Eskildsen, Vladimir S. Fonov, Serge Gauthier, Judes Poirier, Pedro Rosa-Neto

**Affiliations:** 1 Douglas Mental Health University Institute, 6875 Lasalle Boulevard, Montreal, QC H4H 1R3, Canada; 2 Centre for the Studies in the Prevention of Alzheimer’s Disease, 6875 Lasalle Boulevard, Montreal, QC H4H 1R3, Canada; 3 CAPES Foundation, Ministry of Education of Brazil, Brasilia 70040-020, DF, Brazil; 4 McGill University, Montreal, QC H3A 0G4, Canada; 5 Translational Neuroimaging Laboratory, 6825 Lasalle Boulevard, Montreal, QC H4H 1R3, Canada; 6 Departments of Psychiatry and Neurology, University of Pittsburgh School of Medicine, 3811 O’Hara St, Pittsburgh, PA 15213, USA; 7 Montreal Neurological Institute, 3801 University st., Montreal, QC H3A 2B4, Canada; 8 Department of Biochemistry, Federal University of Rio Grande do Sul, Porto Alegre 90035-003, RS, Brazil; 9 Center of Functionally Integrative Neuroscience, Department of Clinical Medicine, Aarhus University, Universitetsbyen 3, 8000 Aarhus, Denmark; 10 McConnell Brain Imaging Centre, Montreal Neurological Institute, McGill University, 3801 University St., Montreal, QC H3A 2B4, Canada; 11 Department of Psychiatry, McGill University, Montreal, QC H3A 0G4, Canada; 12 Department of Neurology and Neurosurgery, McGill University, Montreal, QC H3A 0G4, Canada; 13 The McGill University Research Centre for Studies in Aging 6825 La Salle Boulevard, Montreal, QC H4H 1R3, Canada

**Keywords:** Alzheimer’s disease, mild cognitive impairment, FDG-PET, amyloid PET, memory tests

## Abstract

Associations between pathophysiological events and cognitive measures provide insights regarding brain networks affected during the clinical progression of Alzheimer’s disease (AD). In this study, we assessed patients’ scores in two delayed episodic memory tests, and investigated their associations with regional amyloid deposition and brain metabolism across the clinical spectrum of AD. We assessed the clinical, neuropsychological, structural, and positron emission tomography (PET) baseline measures of participants from the Alzheimer’s Disease Neuroimaging Initiative. Subjects were classified as cognitively normal (CN), or with early (EMCI) or late (LMCI) mild cognitive impairment, or AD dementia. The memory outcome measures of interest were logical memory 30 min delayed recall (LM30) and Rey Auditory Verbal Learning Test 30 min delayed recall (RAVLT30). Voxel-based [^18^F]florbetapir and [^18^F]FDG uptake-ratio maps were constructed and correlations between PET images and cognitive scores were calculated. We found that EMCI individuals had LM30 scores negatively correlated with [^18^F]florbetapir uptake on the right parieto-occipital region. LMCI individuals had LM30 scores positively associated with left lateral temporal lobe [^18^F]FDG uptake, and RAVLT30 scores positively associated with [^18^F]FDG uptake in the left parietal lobe and in the right enthorhinal cortex. Additionally, LMCI individuals had LM30 scores negatively correlated with [^18^F]florbetapir uptake in the right frontal lobe. For the AD group, [^18^F]FDG uptake was positively correlated with LM30 in the left temporal lobe and with RAVLT30 in the right frontal lobe, and [^18^F]florbetapir uptake was negatively correlated with LM30 scores in the right parietal and left frontal lobes. The results show that the association between regional brain metabolism and the severity of episodic memory deficits is dependent on the clinical disease stage, suggesting a dynamic relationship between verbal episodic memory deficits, AD pathophysiology, and clinical disease stages.

## 1. Introduction

Alzheimer’s disease (AD) is the most common cause of degenerative dementia [1,2]. Approximately 55 million people worldwide have dementia and AD accounts for about 60% of cases [1,2]. This high prevalence leads to significant social and economic impact, and it was estimated that the global cost of dementia in 2019 was USD 1.3 trillion [2].

AD has been conceptualized as a process characterized by gradual deposition of amyloid plaques, neurofibrillary tangles, hypometabolism, and neuronal depletion [3,4,5,6]. This pathophysiological process leads to well-known clinical presentation characterized by progressive cognitive decline. 

Decline in episodic memory constitutes the earliest and most prominent cognitive feature in the majority of patients with dementia due to AD [7,8,9,10,11]. Logical memory 30 min delayed recall (LM30) and Rey Auditory Verbal Learning Test 30 min delayed (RAVLT30) are well-known memory outcomes for assessing story recall and word list deficits, respectively, in patients with mild cognitive impairment (MCI) and dementia [12,13,14,15,16,17,18]. LM30 and RAVLT30 abnormalities have been primarily associated with hippocampal memory system dysfunction in AD, although pathological events involving brain circuits subserving memory encoding and retrieval can potentially affect delayed recall [19,20]. 

Previous neuroimaging studies have shown associations between episodic memory, hypometabolism, and amyloidosis in cognitively normal (CN), MCI, and AD patients in brain regions associated with encoding, storage, and retrieval [21,22,23,24,25,26,27,28,29,30]. As such, it might be expected that memory networks could be differentially affected during the clinical phases of AD [8,31,32]. In order to characterize pathophysiological correlations underlying episodic memory deficits in MCI, we adopted a construct from the Alzheimer’s Disease Neuroimaging Initiative (ADNI), which stratifies MCI as early MCI (EMCI) or late MCI (LMCI) according to LM30. It is assumed that EMCI subjects whose deficits in logical memory are less pronounced than those of LMCI subjects represent an earlier stage in the AD spectrum [33,34]. Thus, dichotomization of MCI subjects using this construct may provide a better appraisal of mechanisms underlying memory deficits within the various AD phases. 

The present study aimed to test associations between LM30 and RAVLT30 scores on one hand and voxel-based measures of brain glucose metabolism and amyloid deposition on the other, across the AD clinical spectrum. We hypothesized that we would find associations between the load of AD pathophysiology and memory deficits in brain regions involving memory encoding, storage, or retrieval. Furthermore, we expected to observe stage-specific signatures as well as effects on brain reserve in early AD phases. 

## 2. Materials and Methods

### 2.1. Study Participants

Data used in the preparation of this article were obtained from the Alzheimer’s Disease Neuroimaging Initiative (ADNI) database (adni.loni.usc.edu, last accessed on 13 September 2020). The ADNI was launched in 2003, funded by the National Institute on Aging, the National Institute of Biomedical Imaging and Bioengineering, the Food and Drug Administration, private pharmaceutical companies, and non-profit organizations. The principal investigator involved in this initiative is Michael W. Weiner, MD. The primary goal of ADNI has been to test whether serial magnetic resonance imaging (MRI), positron emission tomography (PET), cerebrospinal fluid, other biological markers can be combined with clinical assessment to measure the progression of AD. The development of sensitive and specific biomarkers of AD progression is necessary for the development of new treatments and to monitor their effectiveness in clinical trials. To date, the four ADNI protocols (ADNI, ADNI-Go, ADNI-2, and ADNI-3) have recruited over 1900 adults to participate in the research, consisting of cognitively normal older individuals, people with early or late MCI, and people with early AD. For up-to-date information, see www.adni-info.org (last accessed on 13 September 2020). 

For the present analysis, we selected all participants from the ADNI-GO and ADNI-2 datasets who were aged between 55 and 90 years old (inclusive) and had completed MRI, [^18^F]florbetapir PET, [^18^F]FDG PET, LM30, and RAVLT30 in the same visit. Individuals were classified as CN, EMCI, LMCI, or AD according to ADNI clinical criteria at the time of the imaging study. Individuals with a modified Hachinski ischemia score higher than four points were excluded during the screening phase, and those with significant vascular changes on structural neuroimaging were excluded from this analysis. 

### 2.2. Classification Criteria

The criteria for CN were absence of memory complaint, absence of cognitive impairment, MMSE scores between 24–30 (inclusive), CDR of 0, and normal memory function as assessed by the LM30 adjusted for education [35]. The criteria for the diagnosis of MCI included the presence of memory complaint, MMSE scores between 24–30 (inclusive), objective memory loss, CDR of 0.5, preserved daily living activities, and absence of dementia [35]. The MCI group was further divided into EMCI and LMCI, based on the LM30 scores adjusted for education. EMCI included individuals with LM30 education-adjusted scores of ≥16 years: 9–11; 8–15 years: 5–9; 0–7 years: 3–6 [35]. LMCI were those with LM30 education-adjusted scores of ≥16 years: ≤8; 8–15 years: ≤4; 0–7 years: ≤2 [35]. Mild AD dementia subjects had memory complaints and abnormal memory function as assessed by LM30 adjusted for education, MMSE scores between 20–26 (inclusive), CDR of 0.5 or 1.0, and fulfilled the NINCDS/ADRDA criteria for probable AD [35,36]. 

### 2.3. Memory Tests

The Rey Auditory Verbal Learning Test and the logical memory test are well-established tests used to assess memory function. 

The RAVLT30 involves free recollection of a list of 15 words, 30 min after 5 immediate recall trials under interference susceptibility [14,15,16]. The score for this test ranges from 0 to 15. 

The LM30 test consists of the recollection of a 25-item short paragraph, measured by the recall of story units after 30 min [17,18]. The score for this test ranges from 0 to 25. 

### 2.4. Imaging Acquisition and Processing

A detailed description of the acquisition of [^18^F]florbetapir and [^18^F]FDG PET images is available at http://adni.loni.usc.edu/methods/pet-analysis-method/pet-analysis/ (last accessed on 13 January 2020). Images were processed as previously described [29]. In summary, T1-weighted MRIs were skull stripped, uniformity corrected, and linearly registered to the MNI space, and deformation fields (4 mm) were calculated for each scan [29,30]. The INSECT algorithm was applied to classify grey matter, white matter, and cerebrospinal fluid, and the ANIMAL algorithm was applied to extract the regions of interest [37]. PET images and their respective MRI images were co-registered using a 6-parameter mutual information algorithm. Individual brain regions were resampled to PET native space. Standard uptake value ratio (SUVR) maps were calculated using the cerebellar grey matter as reference region for [^18^F]florbetapir and the pons a reference region for [^18^F]FDG. Subsequently, [^18^F]florbetapir-PET and [^18^F]FDG-PET images were non-linearly registered to the MNI space using their respective deformation fields. Lastly, images were smoothed with a volumetric Gaussian kernel with a full-width half-maximum of 8 mm [20,38,39]. 

### 2.5. Statistical Analysis

Statistical analyses were performed using R statistical software package version 4.0.2 and RMINC (http://www.r-project.org/ last accessed on 15 September 2020). RMINC is an imaging package that allows analysis of MINC images in the R statistical environment.

Demographics and neuropsychological data were analyzed using ANOVA with post-hoc analysis to perform between-group comparisons. Linear regression modeling was carried out to estimate the associations between cognitive test scores.

Voxel-based linear regressions were performed for [^18^F]FDG and [^18^F]florbetapir SUVR images and LM30 and RAVLT. All regression analyses were corrected for age, sex, years of education, and APOE4 status. The false discovery rate was calculated to correct for multiple comparisons and the corrected threshold of significance was *p* = 0.05 [40]. 

## 3. Results

### 3.1. Demographic and Neuropsychological Data 

Demographics and neuropsychological scores are summarized in Table 1. A total of 452 ADNI participants were included in this study (CN, n = 169; EMCI, n = 134, LMCI, n = 65 and AD, n = 84). The four groups did not differ in sex distribution or years of education, but EMCI participants were nearly 4 years younger than CN and LMCI (*p* < 0.01) and nearly 3 years younger than AD (*p* < 0.05). The EMCI group had lower LM30 and RAVLT30 scores than CN (*p* < 0.01). For the LMCI group, MMSE scores were significantly lower when compared with CN (*p* < 0.01), and LM30 and RAVLT30 scores were significantly lower when compared with CN and EMCI (*p* < 0.01). AD participant scores were significantly lower in all three cognitive tests when compared with CN, EMCI, and LMCI (*p* < 0.01). 

### 3.2. Association between RAVLT30 and LM30

Regression analyses adjusted for age, sex, and years of education showed significant association between LM30 and RAVLT30 (β = 0.655, *p* < 0.001) for all groups combined. When stratified, all diagnostic groups showed significant association between the two cognitive scores: CN (β = 0.317, *p* < 0.001), EMCI (β = 0.442, *p* < 0.001), LMCI (β = 0.564, *p* < 0.001), and AD (β = 0.282, *p* = 0.01). 

### 3.3. Voxel-Based Comparisons of [^18^F]florbetapir Retention and Memory Scores

**Figure 1:**
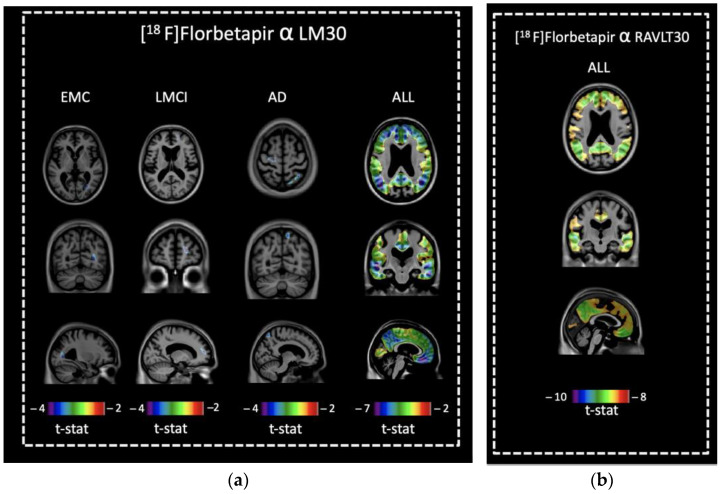
Association between [^18^F]florbetapir retention and memory scores. (**a**) Negative correlations between [^18^F]florbetapir retention and LM30 were observed in the right parieto-occipital region for EMCI, in the right frontal lobe of LMCI, and in the right parietal and left frontal lobes of AD. For all groups combined, negative correlation between [^18^F]florbetapir retention and LM30 was observed in the frontal, parietal, temporal and occipital cortices. (**b**) For all groups combined, negative correlation between [^18^F]florbetapir retention and RAVLT30 was observed in the frontal, parietal, temporal, and occipital cortices.

No associations between LM30 or RAVLT30 scores and [^18^F]florbetapir were observed in CN. In EMCI, LM30 scores were negatively correlated with [^18^F]florbetapir retention in the right parieto-occipital region (Figure 1a). LM30 scores were negatively correlated with [^18^F]florbetapir retention in the right frontal lobe of the LMCI group, and in the right parietal and left frontal lobes of the AD group (Figure 1a). No associations between RAVLT30 scores and [^18^F]florbetapir were observed in EMCI, LMCI, or AD. When all groups were combined, negative correlations between [^18^F]florbetapir retention and RAVLT30 or LM30 scores were observed in frontal, parietal, temporal, and occipital cortices (Figure 1a,b).

### 3.4. Voxel-Based Comparisons of [^18^F]FDG Uptake and Memory Scores

**Figure 2:**
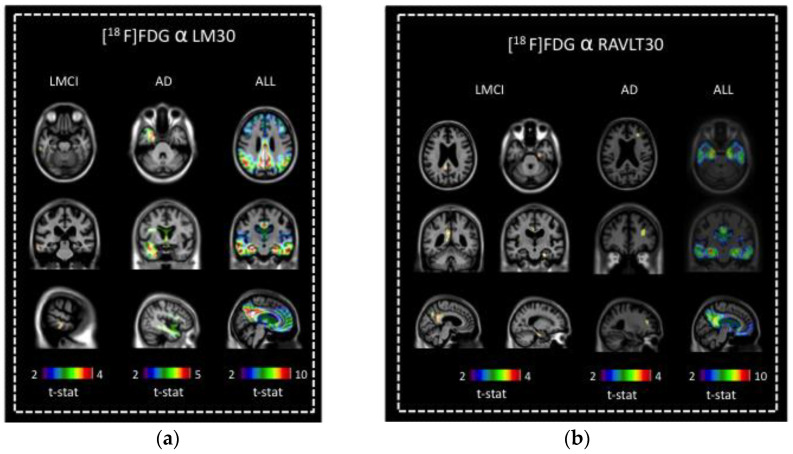
Association between [^18^F]FDG uptake and memory scores. (**a**) Positive correlations between [^18^F]FDG uptake and LM30 were observed in the lateral left middle and inferior temporal gyrus of LMCI and in the antero-medial left temporal lobe of AD. For all groups combined, positive correlations between [^18^F]FDG uptake and LM30 were seen in the frontal and parietal cortices, precuneus, and mesial temporal areas. (**b**) Positive correlations between [^18^F]FDG uptake and RAVLT30 were observed in the left posterior cingulate gyrus and precuneus and in the right enthorinal cortex of LMCI, and in the right frontal lobe of AD. For all groups combined, there was a positive correlation between [^18^F]FDG uptake and RAVLT30 in the frontal and parietal cortices, precuneus, and mesial temporal areas.

No associations between [^18^F]FDG and either of the memory scores were observed in CN or EMCI. In LMCI, LM30 score was positively correlated with [^18^F]FDG uptake in the lateral left middle and inferior temporal gyrus (Figure 2a). In AD, LM30 score was positively correlated with extensive [^18^F]FDG uptake in the antero-medial left temporal lobe (Figure 2a). In LMCI, RAVLT30 was positively correlated with [^18^F]FDG uptake in the left posterior cingulate gyrus and precuneus and in the right enthorinal cortex (Figure 2b). In AD, RAVLT30 was positively correlated with [^18^F]FDG uptake in the right frontal lobe (Figure 2b). For all groups combined, [^18^F]FDG uptake in frontal and parietal cortices, precuneus, and mesial temporal areas including hippocampi and entorhinal cortices was positively correlated with LM30 and RAVLT30 scores (Figure 2a,b).

## 4. Discussion

In this study, we investigated the relationships between episodic memory function and regional amyloid and cerebral metabolism across the AD clinical spectrum. We report that in EMCI, LMCI, and AD, amyloid deposition correlated with LM30 performance in brain areas associated with memory encoding and retrieval. Additionally, we report that hypometabolism was associated with memory decline in LMCI and AD, and that each memory test conveyed a different metabolic deficit in distinct phases of the disease. Overall, our results provide evidence that different memory tests reflect different patterns of brain hypometabolism and amyloidosis across AD clinical phases. 

Voxel-based correlation analysis between behavioral deficits and glucose metabolism or amyloid imaging constitute an effective strategy to explore regional brain dysfunction underlying clinical symptoms caused by AD pathophysiology [20,38,39,41]. When applied to cross-sectional data, this kind of imaging–cognition analysis provides valuable insights regarding localization of pathological processes in slowly progressive neurodegenerative diseases. In this context, an understanding of such imaging–clinical associations can enrich the interpretation of neuropsychological assessments conducted in patients with AD.

LM30 and RAVLT30 are both valid declarative memory outcomes, which reliably describe memory impairment in numerous neurological conditions [42,43,44,45,46]. LM30 and RAVLT30 performance is dependent on the integrity of brain networks subserving memory storage, encoding, and retrieval [47]. Using neuroimaging methods to explore how memory tests reflect pathological damage to these brain networks can improve our understanding of the mechanisms involved in memory decline and also guide our choice of memory assessment in clinical practice.

The present study suggests a dynamic association between delayed recall and AD pathophysiological processes within distinct AD clinical phases, by correlating uptake of [^18^F]FDG and [^18^F]florbetapir with memory deficits captured by LM30 and RAVLT30 in CN, EMCI, LMCI, and AD patients.

### 4.1. Associations of Amyloid Deposition with Delayed Recall

In this study, we found a negative association between amyloid deposition andLM30 in earlier and later phases of AD progression and in areas associated with memory encoding and retrieval, but this was not the case for RAVLT30. 

These findings of a negative correlation between LM30 and amyloid burden in EMCI corroborate previous studies suggesting a link between amyloidosis and delayed recall deficits in early phases of AD [11,20]. Interestingly, the associations found in EMCI involved the parieto-occipital lobe circuits, which possibly subserve encoding or retrieval rather than memory storage [38].

Even though it is known that amyloid load tends to stabilize in LMCI and AD [6,29], the results presented here demonstrate that amyloidosis in relevant areas (parietal and frontal) is associated with memory decline in LMCI and AD, even if only to a small degree. This finding is in line with previous reports that found an association between amyloid load and cognitive decline in more advanced phases of AD [20,38,39].

We report here relevant correlations of memory performance with amyloid deposition in areas associated with memory encoding and retrieval, at different phases of AD development. Because this was a cross-sectional study, we cannot determine whether amyloid deposition directly drives memory decline or whether it is associated with memory deficits through other pathophysiological mechanisms.

### 4.2. Decreased Regional Cerebral Metabolism and Memory

Episodic memory impairment has been reportedly linked with hypometabolism in brain regions associated with encoding, storage, and retrieval, such as the precuneus, cingulate, parietal, temporal, and frontal cortices [28,48,49,50,51,52]. Although previous studies have investigated associations between cerebral metabolism and memory outcome measures, to our knowledge, this is the first assessment of these associations in EMCI and LMCI patients using RAVLT30 and LM30. 

In the present study, we found positive correlations for the LMCI group between RAVLT30 scores and brain metabolism in the left posterior cingulate gyrus and precuneus and in the right entorhinal cortex. For the same diagnostic group, there was a correlation between LM30 scores and hypometabolism in the left middle and inferior temporal gyrus. For LMCI, the low RAVLT30 score reflects hypoactivity in the entorhinal cortex, the first area affected in AD, presumably reflecting depletion of brain reserves subserving memory storage in the earliest stages of the disease. Moreover, hypoactivity in the parietal cortex reflects vulnerability of memory retrieval. The observation that hypometabolism in a smaller area of the temporal cortex correlates with LM30 suggests that the efficiency of context-dependent memory might benefit from brain-reserve mechanisms still present at the LMCI stage.

For the AD group, there was an association between RAVLT30 scores and hypometabolism in the right frontal lobe. For this diagnostic group, the association of hypometabolism and LM30 score was seen in an extensive area in the left temporal lobe, a region involved in memory and language. In line with previous studies, we observed correlations between delayed recall scores and hypometabolism in the AD group, reflecting the dynamic process of brain degeneration in the dementia phase [52,53]. However, in the AD phase we observed a more pronounced correlation of brain hypometabolism with LM30 than with RAVLT30, which suggests higher dependency on contextual memory when the brain reserve mechanisms are further depleted. In this respect, LM30 provides the subject with a context to aid the recollection, while the RAVLT30 is nearly devoid of encoding strategies.

Interestingly, the results of neither test correlated with brain metabolism in EMCI or controls. This suggests a ceiling effect of brain metabolism in earlier stages of the disease, impacting association studies.

Our results are in line with previous studies conducted in AD patients that suggest a stage-specific dynamic progression of hypometabolism [19,25]. Moreover, our findings demonstrate that different memory tests convey different patterns of brain dysfunction in the distinct phases of AD. This difference should be taken into consideration when choosing neuropsychological tests for clinical assessment, and more importantly in research settings and in clinical trials when using neuropsychological tests to evaluate brain impairment across the AD clinical spectrum. 

### 4.3. Limitations of the Study

Interpretation of these findings should take into consideration the following factors. Firstly, the cross-sectional nature of the present study limits the extrapolation of these results in terms of progression of disease pathophysiology. In addition to the exclusion of neurological comorbidities, we excluded participants with high vascular load according to MRI. Nevertheless, we cannot exclude the presence of other etiologies (i.e., TDP-43) underlying MCI or dementia. Although group-specific structural abnormalities were minimized by high-order non-linear registration, it is possible that brain atrophy could confound the interpretation of PET results. Moreover, the fact that LM30 testing was utilized to stratify early and late MCI phases should also be taken into consideration. 

## 5. Conclusions

In conclusion, the present study supports a conceptual framework of dynamic associations between delayed recall tests and brain metabolism, encompassing networks of regions associated with encoding, storage, and retrieval. Indeed, the link between memory performance and regional brain dysfunction was better captured by RAVLT30 in late MCI stages, while LM30 better described the associations in the dementia phase. Most importantly, across the spectrum of clinical presentation of AD, performance in each memory outcome reflects cortical dysfunction across specific nodes within a network of brain areas. This framework proposes a dynamic relationship between verbal episodic memory deficits, AD pathophysiology, and clinical disease stage, as opposed to static associations between memory deficits and the magnitude of cortical dysfunction in specific brain areas. Future research should consider follow-up data to evaluate whether these associations are replicated in longitudinal studies.

## Figures and Tables

**Table 1:** Characteristics of the sample.

	CN (n = 169)	EMCI (n = 134)	LMCI (n = 65)	AD (n = 84)	*p*-Value
Sex (M/F)	86/83	72/62	39/26	50/34	0.463
Age (years)	77.6 ± 6.5	73.6 ± 8.2 ^a^	77.6 ± 7.6 ^b^	76.8 ± 7.3 ^d^	<0.001
Education (years)	16.5 ± 2.7	15.7 ± 2.7	16.2 ± 2.7	16.3 ± 2.6	0.068
MMSE	29.0 ± 1.3	28.4 ± 1.6	27.5 ± 2.1 ^a^	21.4 ± 4.5 ^abc^	<0.001
Logical memory 30 min delay recall	14.5 ± 3.6	11.8 ± 3.7 ^a^	4.1 ± 2.8 ^ab^	1.7 ± 2.8 ^abc^	<0.001
Rey Auditory Verbal Learning Test 30 min delay recall	8.1 ± 4.1	5.6 ± 4.5 ^a^	2.7 ± 3.3 ^ab^	0.7 ± 2.3 ^abc^	<0.001

CN, cognitively normal; EMCI, early mild cognitive impairment; LMCI, late mild cognitive impairment; AD, Alzheimer’s disease; MMSE, mini-mental state examination. All values are indicated as mean ± standard deviation except sex. *p*-value indicates the value assessed with analyses of variance (ANOVA) for each variable except sex, where a contingency chi-square was performed. Post-hoc analysis provided significant differences between groups: ^a^ from CN at *p* < 0.01; ^b^ from EMCI at *p* < 0.01; ^c^ from LMCI at *p* < 0.01, ^d^ from EMCI at *p* < 0.05. Total number of participants: 452.

## Data Availability

All data used in this study can be found in the ADNI repository, www.adni-info.org.
